# Graded Morphologies and the Performance of PffBT4T-2OD:PC_71_BM Devices Using Additive Choice

**DOI:** 10.3390/nano11123367

**Published:** 2021-12-12

**Authors:** Hugo Gaspar, Andrew J. Parnell, Gabriel E. Pérez, Júlio C. Viana, Stephen M. King, Adélio Mendes, Luiz Pereira, Gabriel Bernardo

**Affiliations:** 1 Institute for Polymers and Composites, University of Minho, 4800-058 Guimarães, Portugal; hugogaspar@fe.up.pt (H.G.); jcv@dep.uminho.pt (J.C.V.); 2 LEPABE—Laboratory for Process Engineering, Environment, Biotechnology and Energy, Faculty of Engineering, University of Porto, Rua Dr. Roberto Frias, 4200-465 Porto, Portugal; mendes@fe.up.pt; 3 Department of Physics and Astronomy, The University of Sheffield, Sheffield S3 7RH, UK; a.j.parnell@sheffield.ac.uk; 4 Department of Chemical and Biological Engineering, The University of Sheffield, Sheffield S1 3JD, UK; gabriel.perez-garcia@stfc.ac.uk; 5 ISIS Pulsed Neutron and Muon Source, STFC, Rutherford Appleton Laboratory, Harwell, Oxon OX11 0QX, UK; stephen.king@stfc.ac.uk; 6 Department of Physics and i3N—Institute for Nanostructures, Nanomodelling and Nanofabrication, University of Aveiro, 3810-193 Aveiro, Portugal

**Keywords:** Organic photovoltaics, bulk-heterojunction morphology, additives, PffBT4T-2OD

## Abstract

The impact of several solvent processing additives (1-chloronaphthalene, methylnaphthalene, hexadecane, 1-phenyloctane, and p-anisaldehyde), 3% *v*/*v* in o-dichlorobenzene, on the performance and morphology of poly[(5,6-difluoro-2,1,3-benzothiadiazol-4,7-diyl)-alt-(3,3‴-di(2-octyldodecyl)-2,2′,5′,22033,5″,2‴-quaterthiophen-5,5‴-diyl)] (PffBT4T-2OD):[6,6]-phenyl-C71-butyric acid methyl ester (PC_71_BM)-based polymer solar cells was investigated. Some additives were shown to enhance the power conversion efficiency (PCE) by ~6%, while others decreased the PCE by ~17–25% and a subset of the additives tested completely eliminated any power conversion efficiency and the operation as a photovoltaic device. Grazing-Incidence Wide Angle X-ray Scattering (GIWAXS) revealed a clear stepwise variation in the crystallinity of the systems when changing the additive between the two extreme situations of maximum PCE (1-chloronaphthalene) and null PCE (hexadecane). Small-Angle Neutron Scattering (SANS) revealed that the morphology of devices with PCE ~0% was composed of large domains with correlation lengths of ~30 nm, i.e., much larger than the typical exciton diffusion length (~12 nm) in organic semiconductors. The graded variations in crystallinity and in nano-domain size observed between the two extreme situations (1-chloronaphthalene and hexadecane) were responsible for the observed graded variations in device performance.

## 1. Introduction

Organic photovoltaics (OPV) is an emerging solar cell technology that attracts great interest due to its promising performance and potential for low-cost manufacture, over large areas, on lightweight flexible plastic substrates, which can be fabricated using high-throughput, roll-to-roll (R2R) solution processing. OPVs have undergone rapid development in the last 4 years, with certified power conversion efficiencies (PCE) under AM1.5G conditions improving from 11.5% in 2017 to 18.2% in 2020 [1] for small area devices. These outstanding improvements have caused a surge of research activity in the OPV field towards the transition from the lab to the market [2,3,4]. The future of the OPV technology looks very promising, particularly for small energy production in portable technologies and wearables, when other types of photovoltaics (PV) cannot be easily incorporated (too rigid or too high cost).

The small band gap donor polymer poly[(5,6-difluoro-2,1,3-benzothiadiazol-4,7-diyl)-alt-(3,3‴-di(2-octyldodecyl)2,2′;5′,2″;5″,2‴-quaterthiophen-5,5‴-diyl)] (PffBT4T-2OD), also known as PCE11, exhibits relatively high hole mobility under space charge limited current conditions (SCLC) (over 1.5–3.0 × 10^−2^ cm^2^·V^−1^·s^−1^ [5]) due to its high crystallinity. These properties, together with its tendency to form relatively pure polymer domains when blended with fullerene acceptors, allow it to perform well in an OPV device, when used in relatively thick bulk-heterojunction (BHJ) layers (~300 nm). PffBT4T-2OD has, therefore, been the subject of numerous OPV studies either blended with PC_71_BM [6,7,8,9,10,11,12,13] or with other fullerenes [5,14,15,16].

The processing conditions used in the preparation of a BHJ layer play a crucial role in the formation of the nanomorphology, which is directly linked to the resultant solar cell device performance. The most common processing methodology uses additives to optimize the BHJ morphology and thereby increase device performance in OPV devices based on small band gap copolymers such as PffBT4T-2OD [17,18,19]. Additives can provide fine control of the BHJ morphology by strongly influencing the film formation during solution casting.

Previous studies have shown that additives act primarily during the film drying stage [9] and not in solution [20]. Zhang et al. [9] studied the effect of the additive 1,8-diiodooctane (DIO) on the performance and BHJ morphology of PffBT4T-2OD:PC_71_BM-based devices, using a polymer with *M_w_* = 117,800 g·mol^−1^ and Mn = 54,900 g·mol^−1^. The additive was shown to increase the PCE of the OPV devices by ~20%, from 7.2% to 9.0%, due to a coarsening of the phase domains from an initial characteristic length scale of ~9–10 nm to a final value of ~12–13 nm. DIO was also shown to affect the orientation and crystallinity of the BHJ. The dominant orientation for the blends without additive is a face-on lamellar structure. Annealing this blend for 5 min improved the out-of-plane ordering, as revealed by well-defined peaks for the face-on lamellar packing at 0.29 Å^−1^ along with higher orders at 0.59 Å^−1^ and 0.88 Å^−1^. The sample processed with DIO exhibited a much more isotropic orientation of the polymer crystallites, with Debye–Scherrer -like rings observed instead of the strong out-of-plane orientation scattering features seen without DIO.

Zhao et al. [7] studied the impact of the additives DIO, 1,8-octanedithiol (ODT), diphenylether (DPE), and 1-chloronaphthalene (CN) on the performance of PffBT4T-2OD:PC_71_BM-based devices. The best devices were those processed using CN and displayed an average PCE of 10.01% compared with 7.89% for reference devices processed without additive. DIO and DPE additives both improved the device performance with average PCE values of 8.82% and 9.10%, respectively. By contrast, ODT was shown to have a negative effect on efficiency, producing devices with an average PCE of 7.62%. GIWAXS revealed that DIO and CN affect the molecular orientation of the BHJ films in different directions. CN, which dissolves both PffBT4T-2OD and PC_71_BM, leads to enhanced crystallinity of PffBT4T-2OD in the (100) direction corresponding to the alkyl stacking peak, located at a q_z_ value of 0.29 Å^−1^. By contrast DIO, which dissolves PC_71_BM but not the polymer, has a more profound effect on the crystallization of PffBT4T-2OD in the π-π stacking direction (010). The effect of ODT and DPE on crystallinity was not addressed.

Despite these studies, most device optimizations and choices of processing additive have largely been driven by empiricism. As such, there is still a lack of fundamental understanding of the relationship between the processing additive used, the resultant BHJ morphology, and the corresponding device efficiency. This fundamental understanding has been hampered by the fact that normally only additives that improve performance are reported in the literature. Nevertheless, a deeper understanding of the impact of additives on the BHJ morphology and device performance of OPVs should rely on understanding the impact of additives that both improve performance as well as those additives that have an unfavourable impact and degrade photovoltaic performance. This understanding is required to tailor the desired morphology and device performance properties. As is well known, the final efficiency of a PV device depends primarily on several fundamental properties, from light absorption through to exciton formation and transport and finally to free charge capture at the electrodes. The central stage involving exciton separation and electrical carrier transport plays a crucial role and is critically dependent on the morphology of the BHJ. This is particularly true at donor–acceptor (D–A) interfaces, where exciton separation/recombination occurs via charge transfer states (CTS) and bulk free electrical charge carriers are produced. However, the transport of these free carriers depends on the energy levels acting as carrier traps. All these processes and steps depend on a favourable morphology.

The screening of potential solvents and additives can be performed beyond simple trial and error by using Hansen solubility parameters (HSP) [21]. The HSP are empirical parameters that can be used to predict the chemical affinity between an additive, PC_71_BM, and PffBT4T-2OD. The solubility properties of each solid (polymer or fullerene) and liquid (solvent or additive) can be characterized by their corresponding HSP, which can be pictured as a vector in a 3-D orthogonal space with coordinates δ_D_ (dispersion), δ_P_ (polar), and δ_H_ (hydrogen bonding), where δ_D_ represents its dispersion forces related to van der Waals interactions, δ_P_ represents its polarity related to permanent dipole moments, and δ_H_ represents its ability to establish hydrogen bonding interactions.

In this present work, we studied the impact of five different processing additives (1-chloronaphthalene, 1-methylnaphthalene, hexadecane, 1-phenyloctane, and p-anisaldehyde), with concentrations of 3% *v*/*v* in o-dichlorobenzene, on the BHJ morphology and the efficiency of devices based on the donor:acceptor pair PffBT4T-2OD:PC_71_BM. These additives were selected to have different Hansen solubility parameters [21] and different boiling temperatures. While some of these additives did improve device performance, we deliberately also chose some additives that reduced device efficiency to develop an understanding of the interplay between processing additive chemistry, layer nano-morphology, and device performance.

## 2. Experimental Section

### 2.1. Materials

**Figure 1:**
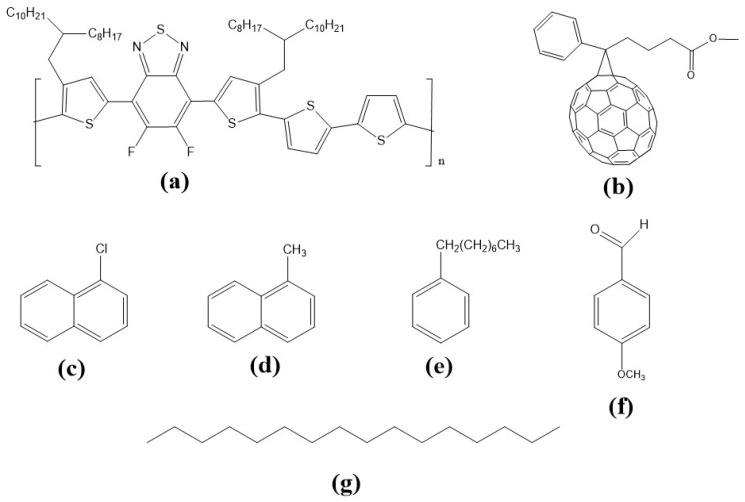
Molecular structures of the polymer, fullerene, and additives used in the present work: (**a**) PffBT4T-2OD; (**b**) PC_71_BM; (**c**) 1-Chloronaphthalene; (**d**) 1-Methylnaphthalene; (**e**) 1-Phenyloctane; (**f**) p-Anisaldehyde; (**g**) Hexadecane.

The following materials were sourced from Ossila Ltd. (Sheffield, UK): (1) Poly(3,4-ethylenedioxy-thiophene):poly(styrene sulfonic acid) (PEDOT:PSS, Heraeus Clevios AI4083); (2) the polymer PffBT4T-2OD (M302) with *M_n_* = 83,008 g mol^−1^ and *M_w_* = 172,033 g mol^−1^; and (3) the fullerene PC_71_BM (M113), [6,6]-phenyl-C71 butyric acid methyl ester, with empirical formula C_82_H_14_O_2_ and *M_w_* = 1030.99 g mol^−1^. The solvent and additives used were all high-purity grade and purchased from Sigma-Aldrich (Gillingham, UK), namely, o-dichlorobenzene, 1-chloronaphthalene, methylnaphthalene, p-anisaldehyde, 1-phenyloctane, and hexadecane. All materials and solvents were used as received without further purification. Figure 1 illustrates the molecular structures of the polymer, fullerene, and additives used in the present work.

### 2.2. Device Fabrication

The standard structure ITO/HTL/Active layer/Ca/Al was used for the OPV devices studied in this work. PEDOT:PSS was used as a hole transport layer (HTL). The active layers were all spin-coated from a solution of o-dichlorobenzene, with 3% of solvent additive (volume percentage) as typically used in this donor:acceptor system, with the polymer PffBT4T-2OD and PC_71_BM having concentrations of 4 mg·mL^−1^ and 4.8 mg·mL^−1,^ respectively (1:1.2 ratio). We note that, contrary to most of the previous work with PffBT4T-2OD, which used solvent mixtures of chlorobenzene:o-dichlorobenzene (1:1) to dissolve the polymer, here it proved necessary to use a pure o-dichlorobenzene solvent due to the relatively high *M_w_* of the polymer used (which had a stronger solubilizing power than the standard chlorobenzene:o-dichlorobenzene mixture). Additionally, we note that all the five different additives were completely miscible with o-dichlorobenzene in the volume ratios (3:97) considered. The active layers were spin-coated from pre-heated solutions (120 °C) at a spin speed of 800 rpm onto the PEDOT:PSS/glass substrate that was pre-heated to 120 °C. The active layer was spin-cast in a nitrogen-filled glove box. The films were then left in the glove box for 2 h to dry and then thermally annealed on a hotplate at 100 °C for 5 min. As a reference, some BHJ films were prepared without additive and were not annealed. The cathode evaporation was then deposited sequentially, first 5 nm of calcium (Ca) and then 100 nm of aluminium (Al) on top of the active layer under a vacuum < 2 × 10^−6^ mbar to form the top electrode contact (cathode). Finally, the devices were encapsulated using UV-cured epoxy (E131, Ossila Ltd., Sheffield, UK) and a glass slide.

### 2.3. Morphological Characterization

Grazing-incidence wide-angle X-ray scattering (GIWAXS) was used to study the impact of the different additives on the crystalline structure of the BHJs. GIWAXS measurements were performed on a Xeuss 2.0 SAXS/WAXS laboratory beamline using a liquid gallium MetalJet (Excillum) X-ray source (9.2 keV,1.34Å). The scattered X-rays were detected using a Pilatus3R 1M detector. Samples were prepared on PEDOT-coated silicon substrates following identical processing to that used in the preparation of actual OPV devices.

SEM and EDS characterizations of the surface of the BHJ films were performed using a desktop SEM Phenom XL microscope.

Atomic Force Microscopy (AFM) was used to image the surface morphology of the PffBT4T-2OD:PC_71_BM BHJ thin films processed with different additives. Analysis was performed with a Digital Instruments, Veeco Multimode NanoScope, in tapping mode using Bruker TESPA-V2 tips.

Small-Angle Neutron Scattering (SANS) was used to study the impact of the different additives on the phase morphology of the BHJs. SANS experiments were performed on the LOQ diffractometer at the ISIS Pulsed Neutron Source (Didcot, UK) and the data were processed according to standard procedures using the Mantid framework (Version 4.0.0, Open Source) and accounting for the measured neutron transmission and thickness of the samples. The SANS data (on an absolute scale) were then fitted to appropriate models using the SasView software (Version 4.1.1, Open Source). For sample preparation, blend films were spin-coated onto 0.5-mm-thick quartz discs (Knight Optical Ltd., Maidstone, UK), pre-coated with PEDOT:PSS following the same procedure used in device fabrication. Therefore, the thickness of the SANS films was exactly the same as the thickness of the device films. Stacks of 16 individual blend films on quartz discs were then assembled in order to produce good signal-to-noise statistics for the SANS measurement, as previously reported [9]. The SANS signal from an equivalent number of quartz substrates covered with PEDOT:PSS was subtracted as a background.

### 2.4. Electrical Characterization

The electrical current density–voltage (J-V) response of the devices was acquired under AM1.5 Global conditions with a Newport 92251A-1000 AM 1.5 solar simulator, which was calibrated using an NREL standard silicon solar cell to ensure an irradiance level of 1000 W/m^2^. To limit the light-exposed area of the device to 2.6 mm^2^, an aperture mask was utilized. A one-diode equivalent electrical circuit was employed for the data simulation using genetic algorithms, as previously reported [22]. Signal analysis (impedance spectroscopy) [23,24,25,26,27] was carried out using an Agilent 4294A RLC Meter in the frequency range of 100 Hz–5 × 10^6^ Hz, with an ac level of 100 mV. The capacitance and loss (conductance/angular frequency) were used to determine the relaxation frequency and the corresponding relaxation time with further correlation with charge separation/recombination at the D-A interface.

## 3. Results and Discussion

**Figure 2:**
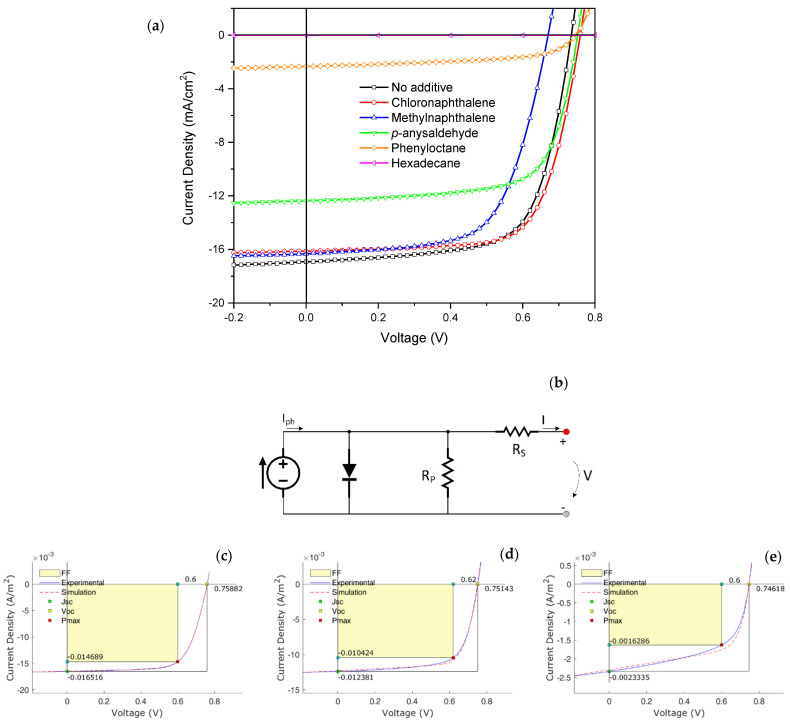
(**a**) J-V curves of devices processed with different additives. (**b**) Equivalent circuit used. Simulations for (**c**) chloronaphthalene, (**d**) p-anisaldehyde, and (**e**) phenyloctane.

Figure 2a and Table 1 present the current density–voltage (J-V) curves and characteristics of devices processed with different additives and a reference device without additives. Figure 2b shows the equivalent circuit used for the simulations, and some example simulation curves are shown in Figure 2c–e for devices with chloronaphthalene, p-anisaldehyde, and 1-phenyloctane, respectively. The corresponding simulation parameters J_ph_, R_s_, and R_p_ are also shown in Table 1. Considering the average values of PCE obtained (first column, inside brackets in Table 1), it can be seen that, compared to the reference device, the additive 1-chloronaphthalene improved the efficiency and the remaining four additives degraded it (methylnaphthalene, p-anisaldehyde, 1-phenyloctane, and hexadecane). Devices prepared using the additive 1-chloronaphthalene exhibited the best overall device performance, yielding an average PCE of 8.44% and a V_oc_, FF, and J_sc_ of 0.75 V, 69.0%, and 14.15 mA∙cm^−2^, respectively. Among the additives that degraded the device performance, 1-phenyloctane and hexadecane were particularly detrimental: 1-phenyloctane reduced the average *PCE* to 0.55% and hexadecane completely destroyed any photovoltaic effect.

The overall comparison of the J-V figures of merit revealed interesting points: All devices exhibited relatively high parallel resistance (higher resistance values indicate lower recombination) and, although a trend could be observed (with higher R_p_ values corresponding to high PCE), this was not the case for OPVs employing 1-phenyloctane and hexadecane as additives. Another relevant parameter was the photocurrent density (J_ph_), as it provides an estimate of the photogenerated electrical carriers. The higher the difference to the J_sc_, the higher was the recombination rate (with the J-V curve being more similar to a pure electrical charge transport under SCLC conditions). In our results, we observed a very significant difference between J_sc_ and J_ph_ when the 1-phenyloctane additive was used (J_sc_ ~0.4 of J_ph_), when the typical value was about J_sc_ ≈ 0.8 × J_ph_ (devices without additives, with methylnaphthalene and chloronaphthalene) and even higher (J_sc_ ≈ 0.97 × J_ph_) when p-anisaldehyde was used as additive. Although the trend was not perfect (and not directly related to R_p_), overall, the best PCE values were obtained in devices with a higher J_sc_/J_ph_ ratio. These results demonstrated that the additives used change the way and how excitons were created/separated at the D–A interface and also changed the electrical charge transport process. Although there were some fluctuations, R_s_ had no noticeable changes independently of the additive used. Moreover, considering the estimated errors associated with the measurements, the deviations in the evaluated values can be attributed to the normal variance observed in the series resistance.

To get more information regarding the exciton separation/recombination at D–A interfaces, some straightforward small signal analysis was performed. Several models are widely used for impedance spectroscopy of OPVs. When devices are measured under dark conditions (as in this case), the data can be interpreted in terms of thickness and position of the layers that form the surface or interface of the device. This means that, typically, two different relaxation processes, besides the simplest charge accumulation/release at electrodes, can eventually be observed and analyzed in the time domain, giving valuable information about electrical charge transport. In a final device, only the slowest electrical carrier diffusion time and its lifetime can be evaluated. These times are related to the time needed for carriers to diffuse out of the bulk layer in the direction of the electrodes and to the recombination time, respectively [28].

**Figure 3:**
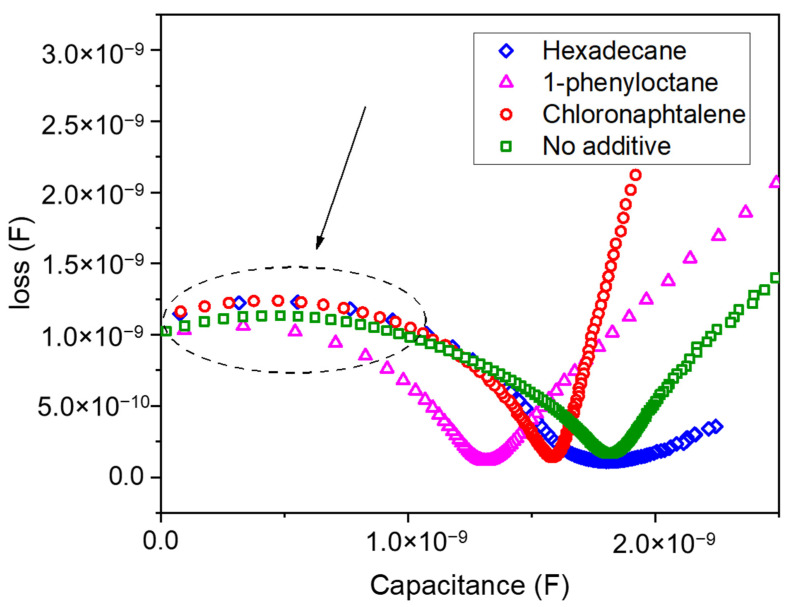
Small signal data of the devices modified with different additives. Nyquist plot enhancing the region of high-frequency relaxation.

Considered as the most representative situations, the capacitance and loss (conductance over frequency, ω) were measured for a reference sample without additives and for samples with hexadecane and 1-phenyloctane (the two most detrimental additives) and with chloronaphthalene (the most favorable additive). The impedance spectra are shown in Figure S1 in Supplementary Information, and the Nyquist plot is shown in Figure 3. From the impedance spectra, it is clear that there were two relaxations, one at low frequency (<1 kHz) and a second at much higher frequency (>2 MHz). The low-frequency range relaxation can usually be attributed to charge accumulation at the electrodes; however, the high-frequency relaxation needs to be more carefully analyzed. From the Nyquist plot in Figure 3, it can be seen that the relaxation peak changed slightly depending on the additive employed. We can determine the relaxation frequency (and, therefore, the relaxation time) for the different measurements. In our case, the relaxation time was near 0.50 µs, 0.53 µs, 0.59 µs, and 0.68 µs for samples with chloronaphthalene and without additive, 1-phenyloctane, and hexadecane, respectively. A simple conclusion was that, as the relaxation time in the high-frequency region increased, we observed a decrease in the PCE, and this was mostly due to a decrease in the J_sc_ (regardless of the relationship with J_ph_). These relaxation times seemed to be correlated to the diffusion time (less than 1 µs) and were counter to the expected slower relaxation times observed for recombination (a few µs). For now, an increase of the relaxation time in our samples corresponded to a decrease of the efficiency that could be linked to electrical transport controlled by electrically active traps, reducing mobility and increasing the diffusion time, leading to a high recombination rate. Some degradation of the D–A interfaces might also have been present, although this was not simple to quantify.

Aiming to elucidate the impact of different additives on the morphology of the BHJ films, we started observing the surface micro-morphology of the different BHJ films, with and without additives, under an SEM. As expected, in the films without additive and with chloronaphthalene, methylnaphthalene, and p-anisaldehyde, the film surfaces were featureless at the micron-scale. However, interestingly, micron-size aggregates could clearly be seen in the films processed with 1-phenyloctane and hexadecane, as shown in Figure S2 in the Supplementary Information. EDS analysis revealed that those aggregates were richer in carbon than the rest of the film surface, strongly suggesting that they were enriched areas of PC_71_BM or possibly pure phases of PC_71_BM.

The impact of the different additives on the surface nano-morphology and degree of phase segregation in the BHJ films processed with different additives were assessed by atomic force microscopy (AFM). A selection of AFM images is presented in Supplementary Figure S3. Although a clear trend can be seen in the root-mean-square (rms) roughness values, which were lower in the film without additives and higher in the films with the most detrimental additive (hexadecane), overall, the interpretation of the AFM images was ambiguous and inconclusive. At this point, it is worth emphasizing that AFM only probes the surface morphology of the film, and this may be very different from that of the underlying bulk material.

**Figure 4:**
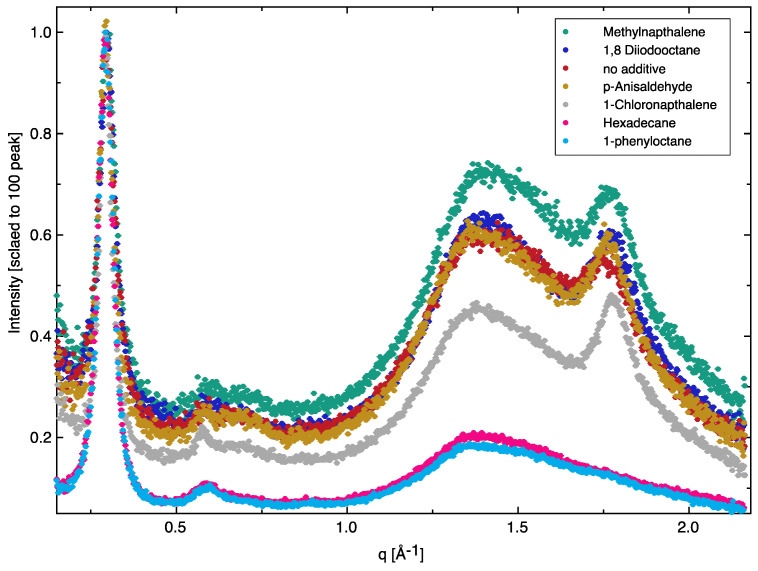
GIWAXS data of the BHJs processed using different additives.

Grazing-incidence wide-angle X-ray scattering (GIWAXS) revealed the molecular packing and crystalline structure of the thin BHJ films processed using different solvent additives. Figure 4 shows GIWAXS spectra scaled to the (100) lamellar peak. The films exhibited a high degree of molecular order, as evidenced by strong lamellar (100) and (200) peaks at 0.29 Å^−1^ and 0.58 Å^−1^ and a very weak (300) peak at 0.87 Å^−1^. More importantly, the (010) peak at q ~1.79 Å^−1^ (corresponding to the polymer π-π stacking) was very pronounced in those BHJ films processed with the best additives and in the film without additives. However, the π-π stacking peak completely disappeared in the BHJ films processed with the most detrimental additives (1-phenyloctane and hexadecane). Table 2 gives more details relevant to the (010) peaks. Additionally, a small shift in the (010) peak position and a change in the full-width at half-maximum (FWHM) was also observed due to the effect of additives. For example, the (010) peak appeared at q = 1.77 Å^−1^ (corresponding to a π-π packing distance of ~3.55 Å) in a film without additives and it appeared at q = 1.78 Å^−1^ (π-π packing distance of ~3.53 Å) in a BHJ processed with chloronaphthalene. A broad halo at q ~1.39 Å^−1^ characteristic of PC_71_BM aggregation could also be observed, which remained almost unchanged in the different BHJs. These GIWAXS results showed clearly that the huge divergence in performance of the devices largely derived from the molecular packing of the semiconductor polymer molecules.

**Figure 5:**
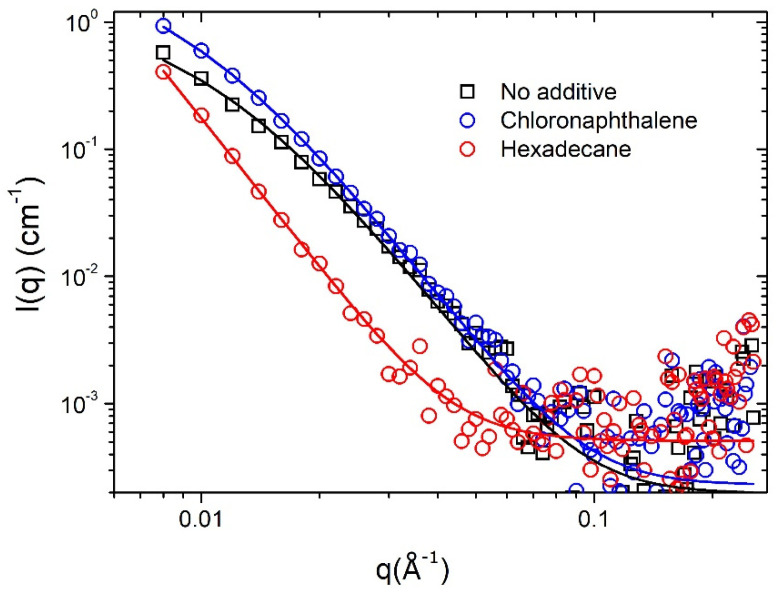
SANS intensity (I) as a function of scattering vector (q) for PffBT4T-2OD:PC_71_BM BHJs processed with 1-chloronaphthalene (best morphology with the highest efficiency) and hexadecane (worst morphology with zero efficiency) and a reference BHJ processed without additives.

Compared to AFM, SANS has the advantage of being a bulk sampling technique that, due to the intrinsic contrast difference in neutron scattering length densities (SLD) between hydrogenous polymers (such as PffBT4T-2OD) and fullerenes (such as PC_71_BM), allows both phase regions to be observed with high contrast, irrespective of them being crystalline or amorphous [29]. In the SANS measurements, we focused on the two extreme cases of additive-modified BHJs, namely, the BHJs processed with 1-chloronaphthalene (highest efficiency) and with hexadecane (zero efficiency), as well as on a reference BHJ processed without additive. In Figure 5, we show the SANS data recorded from stacks of 16 PffBT4T-2OD:PC_71_BM films (corresponding to a total dry active layer thickness of ~3.2 µm). In SANS, the intensity was proportional to the number density, size, and contrast (difference in SLD, Δ*ρ*) of the scattering entities in a sample, while the *q*-dependence of the intensity was related to their shape and local arrangement. Following the same procedure as described in previous work [9,16], the data range from 0.008 Å^−1^ to 0.254 Å^−1^ was fitted using the Debye–Anderson–Brumberger (DAB) (also known as the Debye–Bueche (DB)) model. This describes the scattering from a randomly distributed, two-phase system characterized by a single correlation length, L, a measure of the average spacing between regions of the two different phases. This model has the form:

(1) $$\frac{d\Sigma }{d\Omega }\left(q\right)=\frac{c_{DB}L^{3}}{{\left(1+{\left(qL\right)}^{2}\right)}^{2}}+b$$

where the scaling factor C_DB_ = 8π(Δρ)^2^Φ_1_Φ_2_ between phases has volume fractions of Φ_1_ and Φ_2_. The second term on the right-hand side of the equation (b) is the background intensity that includes both instrumental and sample specific factors, i.e., the incoherent scattering intensity. As shown in Figure 5, the DAB model gives a good description of the data. The values obtained from the fitting for C_DB_ and *L* using equation 1 are given in Table 3. Additionally, also shown are the corresponding values of the reduced χ^2^ that confirm the good quality of the DAB model fits. As shown in Table 3, a BHJ processed without additive had a correlation length of 9.4 nm, whereas the best performing devices, processed with 1-chloronaphthalene, had a correlation length of 11.1 nm. This small coarsening of the phase domains upon the addition of the ‘good’ additives was in agreement with previous reports [9]. By contrast, a BHJ processed with hexadecane (a non-solvent for both the polymer and the fullerene) had a much coarser phase segregation with a correlation length of 31 nm, i.e., much larger than the typical exciton diffusion length in organic semiconductors. Therefore, in a BHJ processed with hexadecane, all the excitons generated will most likely suffer recombination before they are able to reach a D:A interface.

**Figure 6:**
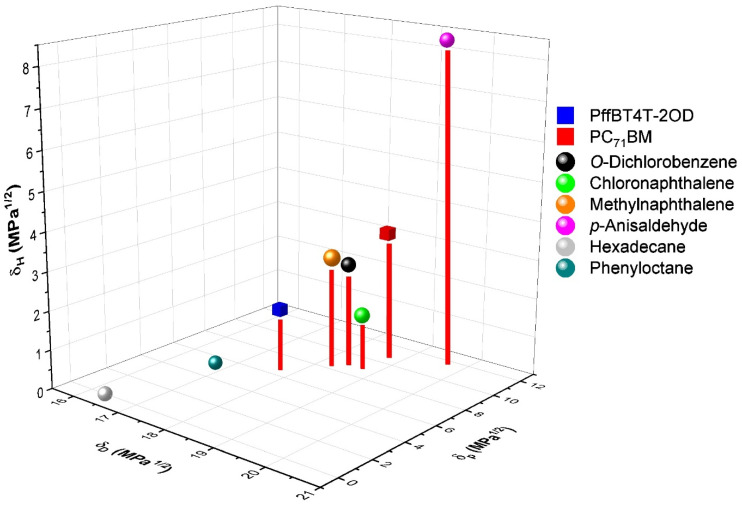
Hansen solubility parameters of polymer, fullerene, solvent, and additives used in this study.

It is well known that, during the spin-coating of the active layer, while the solvent o-DCB (b.p. = 180 °C) evaporates almost completely from the film, the additives largely remain in the film, due to their much lower vapour pressures, thereby slowing down the film drying process [9,20]. Only during the subsequent thermal annealing of the films (5 min in a hotplate at 100 °C), as described in the experimental section, were the additives finally fully removed from the active layer. In this context, Hansen Solubility Parameters (HSP) [21] can help us investigate the correlation between the morphological and efficiency changes caused by the different additives and their chemical affinity for the polymer and fullerene. The Hansen solubility parameters of PffBT4T-2OD [30], PC_71_BM [31], and of the solvent o-dichlorobenzene and the additives [21,32] are shown in Table S1 in the Supplementary Information and are represented graphically in HSP space in Figure 6. It can clearly be seen that additives with HSP values closer to the HSP of PC_71_BM, namely, 1-chloronaphthalene and 1-methylnaphthalene, produced better devices than additives with HSP values that were more distant from the HSP of PC_71_BM. In the case of additives 1-phenyloctane and hexadecane, SEM and SANS analyses demonstrated the formation of large PC_71_BM aggregates both at the micron and nano scales. We hypothesized that this was due to the precipitation of PC_71_BM, promoted by these two non-solvents, and that this occurred during the drying of the active layer films, i.e., post spin coating.

## 4. Conclusions

In this work, we tested the impact of several additives with different solubility parameters and boiling points on the nanoscale morphology and efficiency of bulk-heterojunction solar cells based on the system PffBT4T-2OD:PC71BM. Our results demonstrated the intimate relationship between the power conversion efficiency and the morphology of the devices: (1) the best devices (PCE = 8.75%) exhibited a morphology characterized by a correlation length of ~11 nm and a high degree of π-π stacking and (2) the worst devices (PCE = 0%) exhibited a morphology characterized by a correlation length of ~30 nm, i.e., much higher than the exciton diffusion length, and no π-π stacking. Furthermore, small signal analysis demonstrated that the relaxation time increased when the device PCE decreased. This work demonstrated that the domain size of the PC_71_BM and the degree of phase separation as well as the polymer crystallinity (π-π stacking) are intimately linked to the exciton separation/recombination and the electrical free charge transport and overall solar cell device performance. We showed that this can effectively be predicted and controlled via rational selection of solvent additives using Hansen solubility parameters.

## Figures and Tables

**Table 1:** Device metrics showing the peak and (average) values for PCE, V_oc_, FF, and J_sc_ for devices prepared using different additives. Generated photocurrent (J_ph_) and parallel (R_p_) and series resistances (R_s_) obtained by the equivalent circuit fit with experimental data.

PffBT4T-2OD/PC_71_BM	PCE (%)	V_OC_ (V)	FF (%)	J_sc_ (mA/cm^2^)		Simulation	
					J_ph_ (mA/cm^2^)	R_s_ (Ω)	R_p_ (Ω)
No additive	8.40 (7.96 ± 0.39)	0.73 (0.73 ± 8.86 × 10^−5^)	69.4 (69.0 ± 2.27)	− 14.50 (−13.71 ± 1.16)	17.41	159	3.78 × 10^4^
1-Chloronaphthalene	8.75 (8.44 ± 0.25)	0.76 (0.75 ± 0.008)	70.2 (69.0 ± 1.13)	− 15.09 (−14.15 ± 0.56)	16.50	177	7.65 × 10^4^
Methylnaphthalene	6.98 (6.63 ± 0.19)	0.67 (0.67 ± 0.004)	66.7 (65.8 ± 1.11)	− 13.97 (−13.09 ± 0.50)	16.74	217	5.00 × 10^4^
p-Anisaldehyde	6.46 (5.89 ± 0.53)	0.77 (0.76 ± 0.007)	69.4 (64.5 ± 4.36)	− 12.38 (−11.97 ± 0.37)	12.31	146	4.83 × 10^4^
1-phenyloctane	0.98 (0.55 ± 0.32)	0.76 (0.75 ± 0.008)	56.2 (45.2 ± 8.48)	− 1.63 (−0.98 ± 0.06)	2.29	239	6.70 × 10^4^
Hexadecane	0	-	-	-			

**Table 2:** Details relating to the GIWAXS (010) peaks.

Additive	(010) Peak Position (Å^−1^)	Full-Width at Half-Maximum (FWHM)	π-π Packing Distance (Å)
No additive	1.766 ± 0.002	0.0870 ± 0.0079	3.56
1-Chloronaphthalene	1.781 ± 0.001	0.0588 ± 0.0013	3.53
Methylnaphthalene	1.779 ± 0.001	0.0729 ± 0.0030	3.53
p-Anisaldehyde	1.768 ± 0.001	0.0690 ± 0.0035	3.56
1-phenyloctane	―	―	―
Hexadecane	―	―	―

**Table 3:** Scaling factors (CDB) and correlation lengths (L) obtained by fitting the experimental data using the Debye–Anderson–Brumberger (DAB) model in the interval *q* = 0.008–0.254 Å^−1^.

Additive	Scaling Factor C_DB_	L (nm)	(χ^2^/Npts)
No additive	1.4008 × 10^−6^	9.4 ± 0.1	1.94
Chloronaphthalene	1.993 × 10^−6^	11.1 ± 0.1	1.07
Hexadecane	0.5134 × 10^−6^	31.2 ± 2.4	0.67

## Data Availability

Not applicable.

## Supplementary Materials

The following are available online at https://www.mdpi.com/article/10.3390/nano11123367/s1, Table S1. Hansen solubility parameters of the materials, solvents and additives used in this work, Figure S1. Small signal data: Capacitance and loss as a function of frequency, Figure S2. SEM images of the BHJ films processed using hexadecane as additive, Figure S3. Selection of AFM images of PffBT4T-2OD:PC71BM BHJs processed with different additives.
