# Supplementary material for: Graded Morphologies and the Performance of PffBT4T-2OD:PC71BM Devices Using Additive Choice
Source: Nanomaterials (Basel). 2021 Dec 12;11(12):3367. doi: 10.3390/nano11123367 (PMC8709449; doi:10.3390/nano11123367)
Supplement: Supplementary file 1 [file nanomaterials-11-03367-s001.zip › SI_nanomaterials-1490918.pdf]

nanomaterials

MDPI

# Graded Morphologies and the Performance of PffBT4T-2OD:PC71BM Devices Using Additive Choice

Hugo Gaspar [1,2], Andrew J. Parnell [3], Gabriel E. Pérez [4], Júlio C. Viana [1], Stephen M. King [5], Adélio Mendes [2], Luiz Pereira [6,*] and Gabriel Bernardo [2,*]

**Table S1.** Hansen solubility parameters of the materials, solvents and additives used in this work. To the authors knowledge, no Hansen solubility parameters for p-anisaldehyde have been published in the literature and therefore we indicate the corresponding values for its isomer o-anisaldehyde. We can safely assume that they are similar. (a) Ref. [30]; (b) Ref. [31]; (c) Ref. [21]; (d) Ref. [32].

| | $\delta_D$ MPa$^{1/2}$ | $\delta_P$ MPa$^{1/2}$ | $\delta_H$ MPa$^{1/2}$ | Boiling temperature (ºC) |
|---|---|---|---|---|
| PffBT4T-2OD (a) | 18.56 | 4.07 | 2.31 | -------- |
| $PC_{71}BM$ (b) | 20.2 | 5.4 | 4.5 | -------- |
| o-dichlorobenzene (c) | 19.2 | 6.3 | 3.3 | 180 |
| 1-Chloronaphthalene (c) | 19.9 | 4.9 | 2.5 | 263 |
| 1-Methylnaphthalene (c) | 20.6 | 0.8 | 4.7 | 242 |
| p-Anisaldehyde | -------- | -------- | -------- | 248 |
| o-Anisaldehyde (c) | 19.4 | 11.9 | 8.3 | 238 |
| Hexadecane (c) | 16.3 | 0 | 0 | 287 |
| Phenyl octane (d) | 16.7 | 5.4 | 0 | 262 |

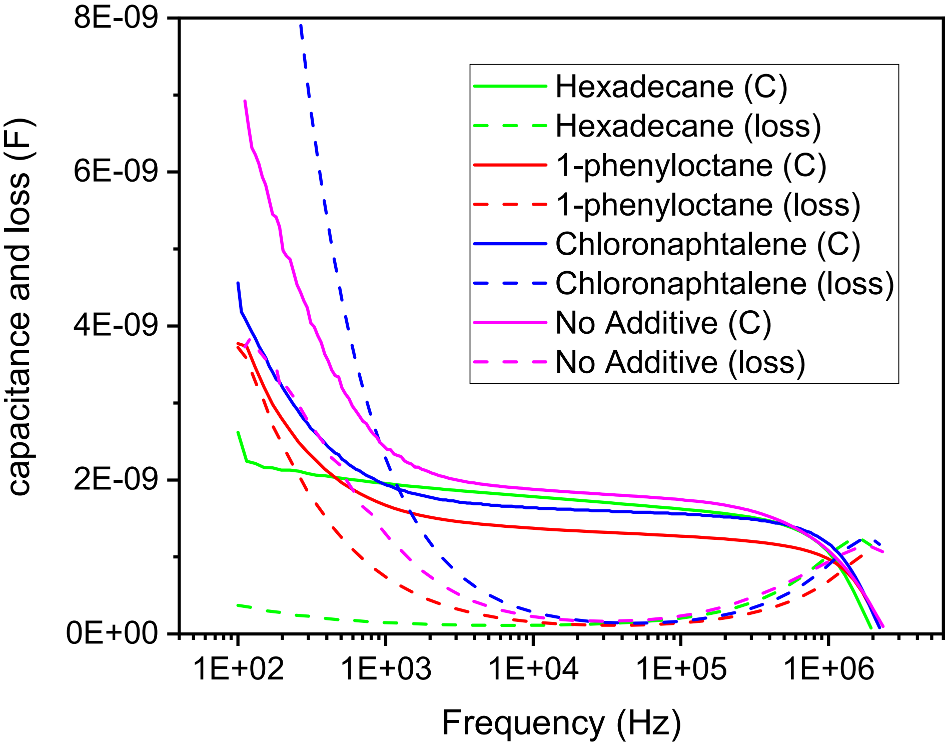


**Figure S1.** Small signal data: Capacitance and loss as a function of frequency.

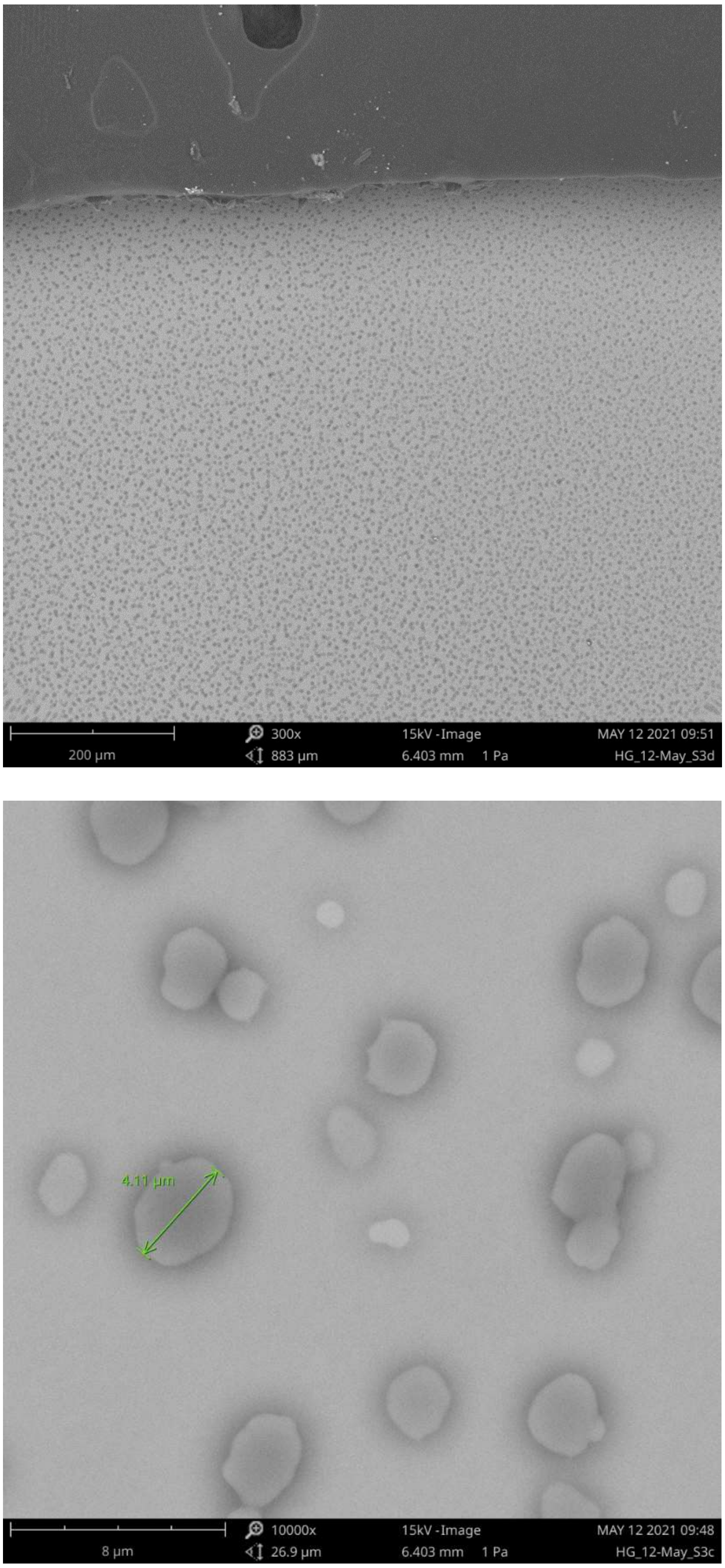


**Figure S2.** SEM images of the BHJ films processed using hexadecane as additive. Micron-size aggregates are clearly visible.

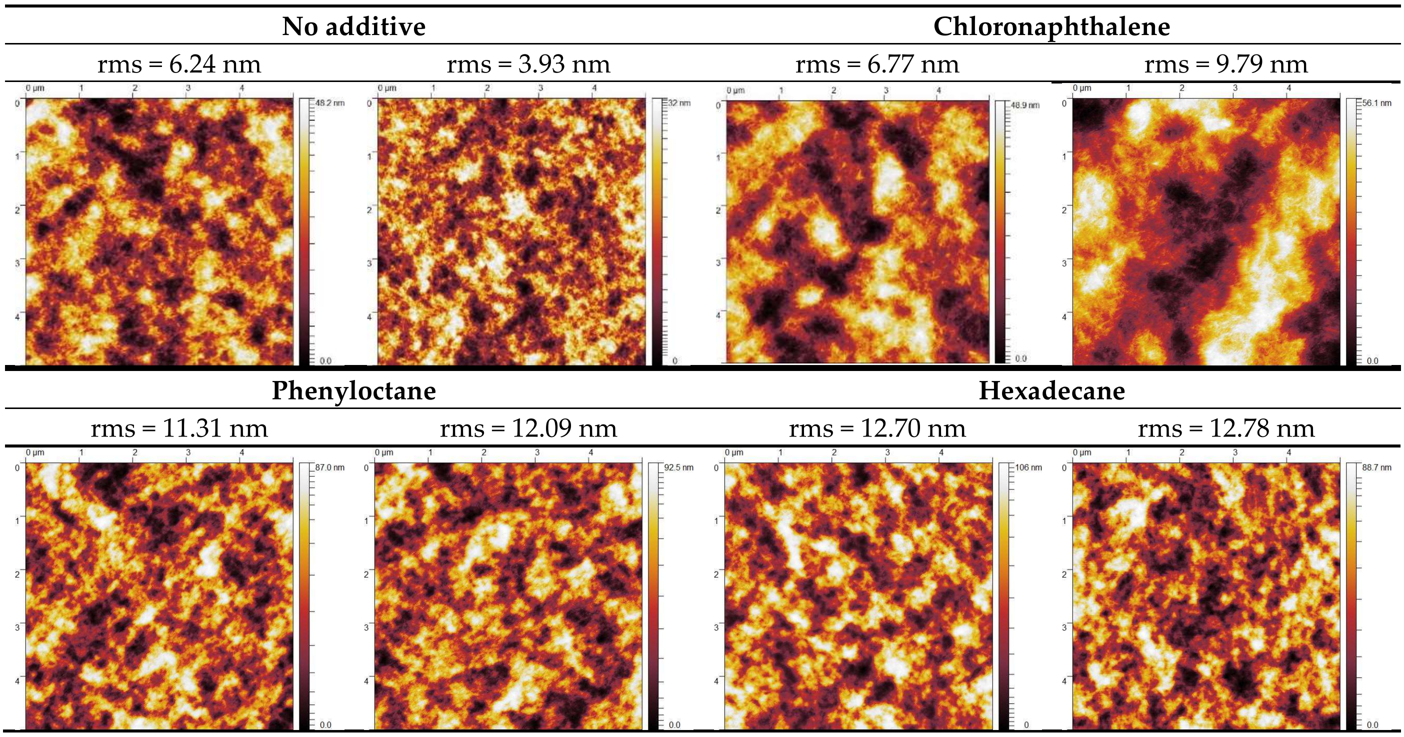


**Figure S3.** Selection of AFM images of PffBT4T-2OD:$PC_{71}BM$ BHJs processed with different additives.
